# Medical termination of pregnancy in general practice in Australia: a descriptive-interpretive qualitative study

**DOI:** 10.1186/s12978-017-0303-8

**Published:** 2017-03-14

**Authors:** Angela J. Dawson, Rachel Nicolls, Deborah Bateson, Anna Doab, Jane Estoesta, Ann Brassil, Elizabeth A. Sullivan

**Affiliations:** 1 The Australian Centre for Public and Population Health Research, Faculty of Health, University of Technology Sydney, P.O. Box 123, Ultimo, NSW 2007 Australia; 2 The Sydney Medical School, Discipline, Gynaecology and Neonatology, University of Sydney and Family Planning New South Wales, 28-336 Liverpool Road, Ashfield, NSW 2131 Australia; 3 Evaluation and Research Operations, Family Planning New South Wales, 28-336 Liverpool Road, Ashfield, NSW 2131 Australia; 4 The Australian Centre for Public and Population Health Research, Faculty of Health, University of Technology Sydney and Family Planning New South Wales, 28-336 Liverpool Road, Ashfield, NSW 2131 Australia

**Keywords:** MTOP, Medical abortion, Mifepristone, General practitioner, Primary health care

## Abstract

**Background:**

Australian Government approval in 2012 for the use of mifepristone and misoprostol for medical termination of pregnancy (MTOP) allows general practitioners (GPs) to provide early gestation abortion in primary care settings. However, uptake of the MTOP provision by GPs appears to be low and the reasons for this have been unclear. This study investigated the provision of and referral for MTOP by GPs.

**Methods:**

We undertook descriptive-interpretive qualitative research and selected participants for diversity using a matrix. Twenty-eight semi-structured interviews and one focus group (*N* = 4), were conducted with 32 GPs (8 MTOP providers, 24 non MTOP providers) in New South Wales, Australia. Interviews were recorded and transcribed verbatim. A framework to examine access to abortion services was used to develop the interview questions and emergent themes identified thematically.

**Results:**

Three main themes emerged: scope of practice; MTOP demand, care and referral; and workforce needs. Many GPs saw abortion as beyond the scope of their practice (i.e. a service others provide in specialist private clinics). Some GPs had religious or moral objections; others regarded MTOP provision as complicated and difficult. While some GPs expressed interest in MTOP provision they were concerned about stigma and the impact it may have on perceptions of their practice and the views of colleagues. Despite a reported variance in demand most MTOP providers were busy but felt isolated. Difficulties in referral to a local public hospital in the case of complications or the provision of surgical abortion were noted.

**Conclusions:**

Exploring the factors which affect access to MTOP in general practice settings provides insights to assist the future planning and delivery of reproductive health services. This research identifies the need for support to increase the number of MTOP GP providers and for GPs who are currently providing MTOP. Alongside these actions provision in the public sector is required. In addition, formalised referral pathways to the public sector are required to ensure timely care in the case of complications or the provision of surgical options. Leadership and coordination across the health sector is needed to facilitate integrated abortion care particularly for rural and low income women.

## Plain English summary

A medical termination of pregnancy (MTOP), also known as an abortion, is a safe and effective procedure to end an early pregnancy. New legislation in New South Wales (NSW) Australia, allows doctors working in General Practice to prescribe medical abortion drugs to end a pregnancy, up to and including 13 weeks. This study aimed to understand the experiences of general practitioners (GPs) working in private practice regarding the provision of MTOP and referral to other health professionals and services. We interviewed 8 GPs who currently provide MTOP and 24 who do not from diverse geographical settings across the State of NSW. Many GPs saw abortion as a service others provide in specialist private clinics. Some GPs had religious or moral objections; others regarded MTOP provision as complicated and difficult. While some GPs expressed interest in MTOP provision they were worried about stigma. Most MTOP GPs were busy but felt isolated. GPs highlighted challenges they had when referring women with complications to local public hospitals or when a woman requested a surgical abortion, particularly for rural and low income women. This research identifies the need to increase the number of GPs who provide MTOP and better support GPs to deliver this service. Formal referral pathways to public health services are needed in the case of complications, or where a woman prefers a surgical abortion. This knowledge is important for planning future reproductive health services that are accessible to all women, regardless of income or place of residence.

## Background

Medical abortion or the medical termination of pregnancy (MTOP) involving the use of abortifacient pharmaceutical drugs has been accessible for early gestation abortions in many countries since the late 1980s and early 1990s [1–3]. The World Health Organization has clear technical guidelines for MTOP methods up to 12 completed weeks [4].

Early MTOP in primary care settings offers women an additional choice to surgical abortion to end an early pregnancy. International evidence demonstrates that MTOP is effective and safe at home and in clinic settings, it has been found to be acceptable to women [5] and cost effective compared with surgical abortion [6]. The introduction of MTOP services alongside surgical abortion has been found to address women’s demand for abortion services, reduce waiting times [6] and improve access for priority populations when provided close to where women live [7].

In Australia, mifepristone along with misoprostol was approved by the Therapeutic Goods Administration (TGA) for commercial import in Australia in 2012, and listed as government subsidised medicine in 2013 [8]. On completion of accredited training General Practitioners (GPs) become certified to prescribe mifepristone and misoprostol in a combination known as MS-2 Step for medical abortion up to 9 weeks gestation in all states in Australia, with the exception of the Northern Territory (NT). In South Australia and the Australian Capital Territory (ACT), MTOP must occur in a licensed facility whereas in other states such as New South Wales (NSW) women are able to undergo an MTOP in their own home [3].

In NSW, women can obtain a surgical or medical abortion at a private clinic in a metropolitan area without a referral from a GP. This procedure will incur an out- of- pocket expense. However, the provision of MTOP in general practice and the public health sector, in addition to private clinics, has the potential to increase women’s access to abortion [9]. As GPs are Australia’s most visited primary care provider [10] they are well positioned to deliver integrated reproductive health care to women that not only includes medical abortion but STI screening, treatment and the provision of contraception. While the cost of the GP consultation for MTOP may be covered by Medicare (the Commonwealth Government’s universal health insurance scheme) for eligible women, GPs may charge a gap payment for their services to cover their costs requiring women to pay the difference as an out-of-pocket expense. There may be additional out-of-pocket costs for women associated with ultrasounds and other tests where Medicare does not cover the providers fee [9]. Mifepristone and misoprostol are subsidised in Australia under the Commonwealth Government’s Pharmaceutical Benefits Scheme. Women who are eligible for a Health Care Card as low-income earners or welfare recipients can receive greater concessions on the price of the medication.

Since TGA approval of mifepristone and misoprostol for medical abortion, 1244 medical practitioners (1.5%) of the 81,478 registered medical practitioners in Australia in 2014 [11] have obtained certification to prescribe. This includes 308 of the 26,112 (1.2%) medical practitioners in NSW [12, 13]. In 2015, the majority of rural and remote areas of NSW had 1-10 MTOP medical prescribers in each of the seven rural/remote health districts in the State (in each of the eight metropolitan health districts there were between 20-40 prescribers) [11, 14]. All together, these health districts serve over one and a half million women of reproductive age [15]. There is no publicly available information regarding which GP practices provide MTOP services in NSW. Little is known about GP MTOP provision, referral routes and workforce issues. One study suggests that uptake of MTOP certification among GPs in NSW is low [14]. Women’s access therefore may be affected by the low number of doctors who provide MTOP due to: low GP knowledge; GP perceptions of high medical indemnity costs, low remuneration, referral challenges and associated stigma; ethical reasons and service priorities [9].

Despite the different jurisdictional legislation, Australians are mostly supportive of the provision of abortion including medical abortion [16, 17]. Traditionally GPs have had a minor role in the provision of abortion care services. The introduction of ambulatory MTOP as a new treatment option for abortion has resulted in some GPs being early adopters. While other GPs may take up MTOP provision in the future there will also be GPs who will refer to other GP providers or services. Therefore, a clear understanding of the context in which GPs provide MTOP or refer to other services is needed to ensure that women requiring abortion services can access them. The aim of this study was to explore the provision and referral of MTOPs by GPs in NSW, Australia.

## Methods

This is a descriptive-interpretive qualitative research study [18] that sought to describe the pathways for women seeking an abortion through general practice in NSW, the factors that determine these from the perspective of GPs, and associated workforce issues. This study was informed by a systematic review of the literature on access to abortion that investigated what is already known about the topic and current knowledge gap [19]. Guided by this approach, we developed open-ended and exploratory research questions, data collection, and analysis strategies which aimed to investigate the nature of GP MTOP provision and referral through the conceptual framework of abortion access described elsewhere [19].

### Selection of study sites and participants: primary health care providers

Purposive maximum variation sampling [20] was used to select GPs in NSW to document the breadth of GP practice so that patterns could be identified that cut across these variations. Stakeholder consultation and service mapping informed the development of a matrix to map the characteristics of primary health care services to guide participant selection. Services in eight areas in metropolitan, regional and remote NSW were selected to reflect community and general practice diversity. The matrix fields included the size of the general practice (sole provider, two-five doctors or more than five doctors); geographical area (Australian Standard Geographic Classification Remoteness Areas: metropolitan, inner and outer regional, remote or very remote), local health district and type and gender of health provider within the service. It was estimated that four participants per town, city and suburb area were required to obtain an appropriate diversity of GP provider characteristics and reveal suitable depth and breadth of data allowing patterns to be revealed [21].

Seventy-two GPs in the selected locations were sent letters and emails inviting them to the study. Practice managers, receptionists and practice nurses were also approached to distribute information to GPs. Recruitment advertisements were placed in the electronic newsletters of local health districts. As recruitment progressed, snowball sampling took place: GP participants provided potential GP contacts, who were invited to take part.

### Data collection

Semi-structured interviews of up to one hour in length were conducted with GPs via telephone or face- to- face in their workplace depending on participant preference. One focus group was held with four GPs. Three sets of open-ended questions to guide interviews were developed by the research team informed by dimensions of access to abortion identified in a previous systematic review [19]. We considered that saturation was reached when no new information or themes were observed in the data.

### Data analysis

Interview transcripts were transcribed verbatim and imported into NVivo10 (qualitative data analysis software package). A thematic analysis was undertaken to identify emergent patterns across and within the transcripts and dimensions [22]. The data was coded by two social scientists (AJD and RN) and a researcher who is also a sexual health nurse (AD). Codes and emergent categories were shared with two medical doctors (ES and DB) and discussed among the group to reach consensus. This provided the inclusion of multiple professional perspectives as well as positions on abortion into the data analysis process. When agreement was reached, coded sections were finalised into categories, and grouped to establish emergent themes and discussed again. Patterns and discrepant themes were explored across the data.

## Results

Twenty-eight individual face-to-face or telephone interviews and one focus group discussion (FGD) with a group of four GPs were undertaken. Table 1 outlines the characteristics of the 32 GP participants in this study.

**Table 1:** General practitioners: Gender, TOP provision status, setting, and area of work

General practitioners		*N* = 32	Percent
Gender	Female	24	75.0
	Male	9	28.1
Role	General Practitioner (GP)	31	96.8
	GP/surgeon^b^	1	3.1
Provision of MTOP	Provider	8	25.0
	Non-provider	24	75.0
Work setting	Private GP practice	32	100
Area (NSW^a^)	Metropolitan	16	50.0
	Inner regional	13	40.6
	Outer regional	1	3.1
	Remote & very remote	2	6.2

^a^Australian Statistical Geography Standard Remoteness Structure [32]

^b^Also known as a procedural GP and defined as a rural or remote GP who ‘provides abortion services, normally in a hospital theatre, maternity care setting or appropriately equipped facility, which in urban areas are typically the province of a specific referral based specialty’ [33]

**Fig. 1:**
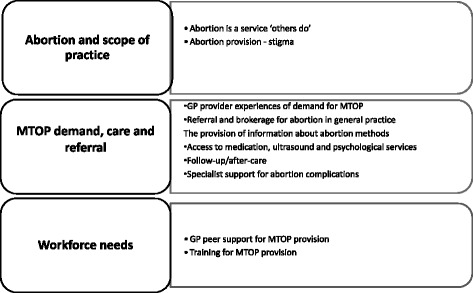
The Key emergent themes and sub-themes

Three main themes were identified with associated subthemes as illustrated in Fig. 1. These are described in the findings that follow.

### Abortion and scope of practice

#### Abortion is a service ‘others do’

Although many GPs believed that it was an essential service, it was not something that they personally wanted to provide:

I remember when I did an anaesthetic term for six months and terminations were just part of the job. I was happy to do it but I remember feeling uncomfortable and it’s a paradoxical thing because I do think that terminations should be freely available but I’m not sure that that’s what I want to make as my business. [GP non-provider, inner regional]

A number of times, both non-providers and providers stated that they did not want to be known as “abortion doctors” and this meant they either were not interested in providing abortion services or were concerned about provision dominating their work. Some were concerned about the negative impact provision may have on their practice reputation and how this might change the family focused practice they aspired to run. Others viewed it as an “unpleasant” service that they did not want to do:

I don’t think I am going to chase that expertise and do it myself ever. I’d rather somebody else handle that. [GP non-provider, metropolitan]

Somebody can do it. I’m not interested. [GP non-provider, metropolitan]

Some GPs also did not want to offer this service because of their personal beliefs. For instance, a young GP pointed out that she did not take up gynaecology as a speciality because as a Muslim she would be ‘uncomfortable’ carrying out abortions:

Life-and-death it’s a big matter for me, so I wouldn’t go into that. [GP non-provider, metropolitan]

There was also a perception among some GPs that there were already existing dedicated private abortion clinics that are able to offer a better service than those working in primary health, primarily because of the abortion provider’s expertise and experience. Therefore, they felt there was no real need for MTOP to be offered through primary practice settings. Some GPs noted that it was “all too hard” to provide MTOP and complicated due to logistical constraints with accessing misoprostol, ordering anti-D immunoglobulin, coordinating ultrasounds and setting up links with the hospital for referral.

#### Abortion provision - stigma

The GPs who provided MTOP tended to be women between 30 to 40 years who were committed to providing comprehensive sexual and reproductive health care and the need to make this service available in primary care. However, this was often in the face of disapproval and judgmental attitudes from colleagues, friends or family. GP MTOP providers noted that they tended to experience subtle judgemental attitudes from others, including strained collegial relationships with colleagues:

Oh, even some of my colleagues… are very set in their views. I found that when they found out that I’m doing these things that they have viewed me differently which is a bit depressing. [GP provider, metropolitan]

MTOP providers were generally reluctant to promote the service over fears of repercussions from those opposed to abortion. One GP had promoted his services through Family Planning NSW, SRH clinics and Women’s Health Centres. Some GP providers were wary of others knowing about their services (including other health professionals) because of potential backlash:

I don’t know how you can ethically advertise it without incurring the wrath of the Christian right wing. [GP provider, inner regional]

GP abortion providers had also experienced negative social consequences from friends. For instance:

My friends that has not been good. I’ve actually had a lot of people be quite negative towards me when they find out that I’m doing this. That’s a very big turn off… I was actually more upset by some of my friend’s opinions. A few of my friends found it very difficult to deal with the thought of me doing these and it took me a long time to actually tell them that I was doing it … they responded more negatively than I thought they would. [GP provider, metropolitan]

Three GP non-providers stated that there was no stigma associated with providing abortion in cases where the provider and woman were not religious or of a faith or culture that supported, or were indifferent to abortion

I don’t know. I’m not religious really. If you are Catholic, Chinese or something maybe. [GP non provider, metropolitan]

One GP also noted that stigma was not an issue when working as a doctor in a setting that focused on reproductive health services for women. Another doctor dismissed stigma as a minor issue compared with the practical challenges of provision.

But I mean that’s not the - the main barrier is actually - yeah, it just wouldn’t work for me from a practical point of view at the moment in terms of - I work for X, I don’t have my own rooms and I have to work within X scope of practice. [GP non provider, metropolitan]

For other GPs, the stigma of being labelled an “abortion doctor” had prevented them from providing MTOP. Other GP non-providers had reservations or refused to provide MTOP because of their personal beliefs.

### MTOP demand, care and referral

#### GP provider experience of demand for MTOP

The GP MTOP providers we interviewed had a range of experience from four months to 2 years.

MTOP GP providers themselves were often unaware of other providers, particularly in rural areas where they felt isolated and unsure if the service was actually needed.

I don’t know whether I’m the only doctor in town who’s on the register…so I don’t know what the demand is for it here - whether I’m one of 10 or the only one. I have no way of knowing. [GP provider inner regional]

Some providers who worked in general practice in both metropolitan and rural areas stated that they had little demand from women for this service, and believed that women were unaware they offered MTOP:

I’ve actually only had two requests since I did the training, and one of them, after the initial visit, went away and thought about it, and changed her mind. So I haven’t had a lot of demand for it … they may be self-referring. [GP provider, metropolitan NSW]

GP interviewees working in dual roles in private abortion clinics and general practice had demand for their services at private clinics but not in general practice. These interviewees had rapidly gained experience in offering MTOP, despite working part-time:

I do about a list of around about 15 to 18 one day a week. I only work one day a week. [GP provider, metropolitan].

Other GPs working in general practice were concerned about being “inundated”, and were increasingly seeing more women requesting MTOP who had heard about their services “through the grapevine” and via “word of mouth”, online forums or social media. Many GPs who were seeing women from out of their practice area stated that they were planning to limit the number of women accessing their services because they were concerned about their capacity to cope and MTOP provision dominating their practice.

Some GP MTOP providers were motivated to provide MTOP primarily because they saw a demand from Indigenous and low income women and stated that there was a need to improve access for these groups who faced barriers relating to cost and transport. One GP provider spoke about the importance of MTOP for Aboriginal women:

I think it’s certainly a more accessible option for them because it doesn’t have a financial barrier or a distance barrier, because the other thing about the Aboriginal population is if they are a pensioner and we prescribe a medication on the PBS it’s actually free altogether [GP provider, outer rural].

Some GPs wanted to offer MTOP but stated that the practice would not permit it.

I don’t provide it in my current work places because it is not supported by my employers. I guess I’m a little bit restricted in that two places where I work… it is currently deemed outside the scope of practice. So I need my employer to accept that it’s within the scope of practice. [GP non provider, inner rural].

*A*ccording to GP participants, offering MTOP in general practice settings can be challenging and time consuming. Although one GP found the provision of MTOP in general practice very straightforward, most GP providers felt that integrating MTOP into their practice involved much time, thought and preparation. They were presented with new challenges, including more patient visits, longer patient counselling time and needed to spend time consulting with local pharmacists and establishing referral pathways in the event of complications.

#### Referral and brokerage for abortion in general practice

GP interviewees said they usually referred women to private abortion clinics. They also referred women to gynaecologists in private practice and occasionally to public hospitals if their patient was facing difficult social circumstances or were adolescents, considered low income, or in cases of sexual assault. GPs in regional areas often felt that women usually self-referred to clinics in the cities. Among GP non-providers there was uncertainty about which GP’s provided MTOP and therefore who to refer to:

There’s rumours about a prescriber for medical in town but I don’t know if they’re true or not. [GP non-provider, inner regional]

All the GP non-providers interviewed stated that they referred women to abortion services, even if they were personally opposed to abortion. Opposition was usually based on religious beliefs:

I am a Muslim so abortion is not recommended unless it is endangering the mum’s life. Around the 120 day, umm… Is it 120 days?… I’ve forgotten that specific number, but we believe that at that specific time the soul is breathed into the foetus, and if it’s done before that that’s not so bad. If it’s done after that, it accounts to murder. That’s how my belief goes, and I couldn’t let go of that. But I’m happy to talk to woman about it and refer to someone who can do it for them [GP non-provider, metropolitan]

One interviewee reported that she delayed referring women so that they could have more thinking time:

Letting them know that they’ve actually got time in many situations to make a decision. It’s not a decision that needs to be made straight away. I think that to me is so important. Any decision that is made at that point has the potential of affecting them forever… it’s just whether they do go ahead with the termination or they don’t go ahead with the termination, there are consequences either way. That to me in that first consultation is so important. We’ll walk through this together making sure that they’re safe at that moment in time for them to go away and digest everything that was said and then coming back for review and follow up. [GP non-provider, metropolitan]

If the patient was low income, GP interviewees noted the lack of opportunities available for referring women for publicly funded abortions through the hospital system. One regional GP recounted a story of a 15-year-old intellectually challenged Aboriginal patient who was pregnant as a result of rape and was requesting an abortion:

Eventually I got one from… one of the obstetricians here. I first of all had it declined and then I rang them up and it was only because I started crying that he agreed to do the termination because he’s known me for a long time. He basically sort of said oh, for God’s sake …, I’ll do it, but I’m not doing it again. So you can imagine how difficult it must be for women themselves without an advocate like you trying to access them. [GP, non-provider outer regional]

GPs in rural areas said that they referred women on to public hospitals only in “extenuating circumstances”. Few metropolitan GPs reported that they attempted to access the public system for their patient and many were not aware this may be an option. For the hospital to accept their patient, the GP had to have an established relationship with the hospital providers, and needed to be able to provide clear justification. They also noted that they had to be careful not to ‘overwork’ public providers. One regional GP interviewee said that she was able to refer one person every two years to the public hospital but the arrangement is not formal and was based on “goodwill”.

Some GPs expressed confusion over appropriate places to refer their patients:

I’m starting to question myself about whether I know all the possible referral avenues with regards to abortion. It’s something I need to just go over, it might just be today. I think just having that information and having the right information and the appropriate information that we can pass on to our clients. [GP non-provider, metropolitan]

One GP interviewee working in a sexual health clinic said that she had managed to get a private clinic to assist with Medicare bulk billing a low-income patient but she said this was really quite unusual:

Possible but rare, and often that’s also a matter of exhausting all other avenues of loans and brokerage from women’s health services. [GP non provider, metropolitan]

#### The provision of information about abortion methods

All the GPs interviewees said that they informed their patients about both medical and surgical abortion methods before a final decision was made:

Oh, we go through the phases of procedures in detail and then it’s up to them. What exactly is going to happen, what the side effects will be with both and we just allow them to decide what they would feel is more convenient for themselves. [GP provider, metropolitan]

Those GPs offering MTOP stated that they referred women on to their colleagues or an abortion clinic for a surgical abortion, if this is what the patient requested. The choice of either method was reportedly based on the preferences, needs and circumstances of patients. However, GPs reported that most of their patients did not ask about medical abortion because they were not aware of this procedure or because they were unaware some GPs provided this service:

I guess because there’s limited experience with medical terminations, most of them haven’t really asked; they just assume that it’s surgical. [GP provider, metropolitan]

One GP interviewee was concerned that the low knowledge of medical abortions among women would mean that women may not be able to advocate for themselves. Thus, women may not be offered a choice:

She had a termination on Tuesday, she had it in [a town nearby] and she was only five weeks and six days and I’m surprised that they actually did a surgical termination for her …I would have thought she would have been a classic example of someone who would benefit from a medical termination myself. But, she wasn’t offered one. [GP provider, regional]

GP uncertainty or limited knowledge of abortion was a feature of a small number of interviews with GPs. More than one GP non-provider was unsure about whether the emergency contraceptive pill was considered an abortion and interviewees occasionally expressed uncertainty about the legality of abortion in NSW.

#### Access to medication ultrasound and psychological services

GPs who worked in dual roles as community GPs and MTOP and surgical abortion providers in abortion clinics had practical experience of the difficulties establishing procedures and accessing supporting services such as ultrasound in general practice settings. It was considered more efficient to refer women on to abortion clinics as they were seen to have all the needed medication, equipment and protocols in place, in case of complications:

Working at (an abortion clinic which provides MTOP) has been really easy. I tried once to offer it to one of my patients in my general practice and that just ended up being too hard. I couldn’t find anywhere to get the medication from and the pharmacists weren’t prepared to get accredited because it would be very - oh they’d do it, but it would take too long to get accredited. [GP provider, metropolitan]

In general practice, GPs had found it, or perceived it to be, difficult to organise an ultrasound in a timely manner and order anti-D immunoglobulin for women who were Rhesus negative. In rural areas where ultrasounds are only available at hospitals, this sometimes proved to be difficult. One GP provider found that asking local gynaecologists in the public hospital to perform ultrasounds on women is:

Asking them for a favour…which they’ve already stipulated that they don’t actually want to do. [GP provider, regional]

However, most concern was expressed over challenges in accessing medication. A GP working in a rural area felt that she wouldn’t consider asking her local pharmacists to stock MTOP medication because they currently refuse to provide the morning after pill while another GP described supply issues incorrectly believing that it is expensive for pharmacists to hold abortion medications:

That proved to be a bit of a challenge when the one patient I had wanted to get it, that we had to hunt it down and the chemist didn’t have it in stock, and it was a little bit of a thing, and I guess if there’s not that much demand for it, that’s going to continue to be a problem, because if they only have a need for it once in a blue moon they’re not going to keep it in stock, so it’s going to be something tricky to get for the patient. [GP provider, outer rural]

Further, it was noted that not all pharmacists working in a pharmacy are registered to prescribe mifepristone and misoprostol:

If the right pharmacist wasn’t on at the time when the person came to purchase the medication they couldn’t get it. So I picked one particular pharmacy where I knew all of the pharmacists had done the training and so I specifically direct my patients to one of two pharmacies and then say if they don’t have it or can’t get then I ask them to call around and I expect that they would do that I think. [GP provider, inner regional]

The pharmacist’s beliefs may also be a barrier. One GP reported that her local pharmacist eventually agreed to dispense, but:

Had to go away and have a bit of a think about whether they wanted to be involved [GP provider, metropolitan]

Interviewees felt it was necessary to establish a good working relationship with a pharmacist who is willing to register for dispensing medications. This takes time and health providers mentioned that alternative pharmacists needed to be found if local pharmacists were not available and incorrectly believed that pharmacists had to receive training.

It was sometimes necessary at the request of women to find an experienced psychologist or counsellor nearby, to provide appropriate and timely pregnancy options counselling or psychological support if needed*.*

They [psychologists] need to be amendable to seeing a client within a week as there’s time constraints. [GP provider, metropolitan]

#### Follow-up/after-care

Adequate follow-up of women post- abortion was a key issue that was raised numerous times by both providers and non-providers. More than one GP provider was very concerned about losing patients to follow-up:

We have a huge dropout rate. We have a huge amount of people that don’t come back. Whilst we always try to phone them and send them emails and things sometimes the phone number’s not correct. I find it quite stressful. I find that more stressful than anything else, not knowing… [GP provider, metropolitan]

One of the main concerns of GPs appeared to be that women who have medical abortions are more likely to be lost to follow-up. Although medical abortion provision was seen as potentially improving access, surgical abortions were seen as more of a ‘safe complete option’ on the day, and women were more likely to return for follow-up:

They’re more likely to come back afterwards I find for follow up just because it’s more of an operation or a procedure that they may feel need follow up for rather than taking a pill. [GP non-provider, regional]

Urban women who travelled long distances to access low cost MTOPs from GP providers were said to be more unlikely to return for follow-up:

My biggest problem is that I have women travelling …to access a termination because I will provide it very cheaply or bulkbilled for healthcare cardholders… Despite all of my best efforts, I am still having great problems getting people to come back for follow up. [GP provider, metropolitan]

GP providers were more comfortable providing MTOP to regular patients who lived locally so that they could ensure proper follow-up and contraception provision. For these reasons, having an established relationship with patients was valued:

I was able to organise the medical termination for her and then she came back in and had her IUD inserted. It was all a very nice - as though it was part of a holistic care package. I was able to manage that complication for her with a good understanding of her family circumstances and her own. I think it did work very well for her …because obviously I had ongoing contact with her and was able to follow up with her how she felt about everything. [GP provider, metropolitan]

If the patient did return for follow-up, GP providers were also worried that they would be unable to access an ultrasound for their patients. GPs were usually only able to refer women to private ultrasound services that were not open past traditional business hours. GPs had found it challenging to obtain emergency ultrasounds through private providers and emergency departments at public hospitals were seen as inappropriate, primarily because of privacy concerns.

GP providers were also concerned that patients who failed to return for follow-up were unable to be provided with contraception. There were also GPs who said that they felt overwhelmed by their workload and were unlikely to offer MTOP because of the possibility of after-hours care:

Because it’s got after hours and all. You can’t cover everything. [GP non-provider, metropolitan].

A GP-surgeon who provided surgical abortions in a rural public hospital had decided not to provide MTOP because of the follow-up required. As many of his patients travelled to access this free service, from both urban and rural areas in the state, he felt that it would be too difficult to ensure appropriate follow-up for patients. Surgical provision was regarded as more straightforward as it does not require routine follow-up:

It’s not a matter of just giving a pill. There’s quite a lot of follow-up for people that are coming from all over the place. I just thought that would be unwieldy for me to try and do that… I work almost exclusively now doing surgery. So I was happy just to offer a straightforward surgical service. But if they want medical terminations they need to go elsewhere. [GP provider, inner regional]

#### Specialist support for abortion complications

It was recognised by some GPs that they needed good contacts with a referral service: “a friendly gynaecologist” in a public hospital who would look after any complications. However, there were several reports by health professionals that public hospitals were reluctant or unwilling to be involved. For instance, a rural GP provider who had sought specialist obstetrician/gynaecology intervention for a MTOP patient who had retained products was unable to have a curette at her local hospital because the gynaecologists refused to do so. This GP no longer performs MTOP because of the lack of support from local gynaecologists:

I know that my colleagues were concerned that if a woman was bleeding a lot that there needs to be support of the gynaecologist that can basically be on call or they can get help quickly, and they didn’t feel that that would be possible. [GP ex-provider, regional]

### Workforce needs

#### GP Peer support for MTOP provision

GP MTOP providers discussed gaps in peer support mechanisms to enable continuity of care and professional development. That may pertain to the new practice environment rather than associated stigma. Within the practice, appropriate documents, policies and procedures need to be developed, particularly those relating to the follow-up and consent of patients. The MTOP GP providers interviewed were mainly females with young children who worked part-time. They tended to work as the sole MTOP provider in their practice and were therefore concerned about follow-up on days they were not working. Support from colleagues to offer follow-up was therefore an important consideration:

It would be good to have support of colleagues that you know can look after things on their day in the clinic when you’re not there (GP provider, metropolitan].

GP providers who were the sole providers of MTOP in their practice were worried that they would not have the support of colleagues if clients needed follow-up on a day they weren’t available. One GP reported that his colleagues were willing to continue providing back-up for all patients, except those that had an abortion. This meant that they had to come in to work to attend to their patients.

One issue with MTOP GP provision centred on challenges building the necessary experience to adequately care for MTOP patients. GPs who worked in dual roles in general practice and private abortion clinics where they provided the bulk of their MTOP services, argued that it is very challenging to provide medical abortions unless it’s something done regularly. In general practice, GPs may not see many MTOP patients and it would therefore be difficult to build necessary expertise:

I think it’s a huge barrier for people doing it out in the community and, look, I’m trained and I know I can do my ultrasounds and for me to do it in the community would be a lot easier probably than a lot of the others. Because at least I know what I have to do to get myself set up to do it. I still found it very, very challenging that one time I tried to do it. In the end I gave up and sent her to [sexual and reproductive health clinic] anyway. I just booked them in on the day I was there rather than trying to get it for her in the community. [GP provider, metropolitan]

Some GPs MTOP providers felt isolated and unsure and felt there was a need for more senior supervision of inexperienced doctors. For example:

I’m kind of a young doctor and doing it all by myself without any support of a supervisor, it’s difficult. So yeah, having more senior doctors who can give more of the termination supervision. [GP provider, regional]

One provider felt she needed to have experienced providers to talk through the online certification training with. The lack of support was also raised by other less experienced GPs:

I’m not ready at this point. I’m a young doctor so I want to get more confidence but who is going to mentor me? [GP non-provider, regional]

#### Training for MTOP provision

The certification training course was positively received by some GP participants. However, a few were concerned about the time it took to do which ranged from 2 to 15 h despite the curriculum indicating four hours. Some GPs felt that they did not require any training support but there was also recognition that in order to provide medical abortions, they needed to update or learn new skills, despite being so time poor:

I am not too sure how RU86 works or how methotrexate is given. I’m sure there is a protocol for it…To be able to answer some questions or if you’re worried about contraindications or aware of not a very typical scenario for example. [GP non-provider, regional]

An inner regional based GP believed that some communications skills training would also be useful. There were also calls by one MTOP provider for more information on where to report adverse events and where to access after- care advice if complications arise.

Sensitivity training for reception staff to ensure they are non-judgemental and maintain women’s privacy was seen as important to GP MTOP provision. In addition, GPs felt women were unlikely to disclose to the reception staff why they wanted an appointment that could delay especially if waiting lists were long due to a shortage of GPs in rural areas.

They’re not likely to tell that to the reception staff, so they’ve got to have some acceptable entrée… Often people use I’ve got tummy ache or something. Tell me about your tummy ache: ‘I haven’t got tummy ache, but I didn’t like to say anything’. So again that’s a barrier, they’ve got to have the nouse to think up to get an entry [GP provider, rural]

## Discussion

Our qualitative study provides the first insight, from the perspective of GPs across the state of NSW, into the factors determining GP MTOP provision/non-provision and the issues concerning the referral of women to other GPs for MTOP and referral to hospitals in the case of MTOP complications or for surgical abortion. These insights provide important information about the strategies that are required to enhance women’s access to MTOP in general practice and improve referral routes.

### Engaging GPs and supporting MTOP provision in primary care

Our research found general agreement that abortion is an essential service which is consistent with recent Australian research from the state of Victoria [23]. However, most of the GPs interviewed did not want to be a MTOP provider and felt that it was a stigmatised service. The GP MTOP providers in our study (25%) were motivated to provide MTOP to assist rural and low income women for whom access to abortion was particularly challenging. However, the GP participants in our study who did not provide MTOP (75%) regarded abortion provision in private clinics to be preferable to general practice, as these clinics were perceived to provide specialised high quality service. These views and GP perceptions and motivations for becoming an MTOP provider may be related to a poor understanding of the processes involved, affecting GP MTOP provision.

Improving GP knowledge of MTOP may help to encourage GPs to become certified providers. It is not known how aware GPs are of the MTOP credentialing course or their intentions/ interest in provision in NSW or beyond. Further marketing of the MTOP training course may be needed alongside professional opportunities for GP MTOP providers to outline the processes and share quality care experiences with fellow GPs, such as integrated contraception care post MTOP identified in our study.

There may also be a need to increase knowledge and awareness of abortion in the pre-service context. Indeed, we know little about the attitudes and intentions of medical students towards abortion training and provision, which would provide insight into the future supply of MTOP GP providers. Generating awareness of these options and training within the professional colleges including the Royal Australian College of General Practice (RACGP), Royal Australian and New Zealand College of Obstetricians and Gynaecologists (RANZCOG) the Australian College of Rural and Remote Medicine (ACRRM), through accredited Family Planning training organisations and in medical and pharmacy schools may also assist in improving GP MTOP provider supply, as has been suggested as a way forward in Canada [24].

In our study, some GPs indicated an interest in providing MTOP but were concerned about stigma and the perceptions of other colleagues if they proceeded. Education and advocacy including GP champions may be helpful to support GPs to engage in discussions about MTOP provision with colleagues and partners of the practices they work in. Such discussion and education may pave the way for an increase in GP MTOP provision.

GP MTOP providers in our study described feeling isolated, stressed and lacking the input from experienced providers. Other research has identified that newly trained physicians often lack the professional support and autonomy necessary to offer abortion services [25]. This highlights the need for mentors and a network of GP providers who are able to work together to ensure follow-up including counselling for contraception. Such peer support could be led by the RACGP and Family Planning training organisations and integrated in practice protocols at the individual, local health district level.

GPs in our study said that they did not provide MTOP because they did not want to experience the stigma and discrimination that is often associated with abortion provision nor change community perceptions of their practices. Various strategies have been explored at a professional level to reduce the burden of stigma. This includes workshops in America where providers shared stories that resulted in the building of interpersonal connections, resilience and collective identify [26]. These might be useful strategies in Australia. Sensitivity training for reception staff may also be a useful strategy and has been included as an indicator in reproductive health service evaluations [27]. While GPs have a right to conscientious objection, this highlights the need for robust referral systems and a whole of health system approach in NSW to ensure women receive timely referral and access to abortion services without incurring financial hardship.

### Improving referral for abortion

In our study, referral was largely affected by the ability GP’s to access up- to- date information and resources which could potentially impact upon the timeliness of abortion and women’s options. GPs identified that there are no formal abortion referral pathways and were largely unaware of GP MTOP providers or public hospital services that provided abortion. This highlights the need for GP peer networks and information that strikes a balance between publicly available information and privacy concerns to improve referral pathways from GP non providers to GP MTOP providers and to a public hospital if required. In line with other studies, GP MTOP providers also noted the need to develop key relationships with local social workers, psychologists, women’s health services, pharmacists and ultrasound providers [23, 28].

We found that public hospital referral for abortion or the management of post abortion complications was particularly challenging and contingent on a prior professional relationship with a gynaecologist. This indicates a need to strengthen not only professional relationships but to address structural issues to formalise pathways for referral to public hospitals and establish systems to provide both timely surgical and medical abortion. This is particularly important for low income women and women living out of cities who cannot afford or travel to, or pay for services at private abortion clinics and for whom public provision may be the only option. These structural deficits serve to provide further evidence for the need for a more focused debate on factors that impinge upon the ability of doctors to provide abortion and strategies to integrate abortion into practice, rather than a discourse that concentrates on the need to protect doctor’s right to conscientious objection [29].

A publicly funded pregnancy advisory service similar to the service provided in South Australia [30] may provide a model in local area health networks where women in NSW could access both medical and surgical abortion, pregnancy options counselling, contraception services and information. This would provide GPs with a central referral point for women to access comprehensive abortion care. Such a service could serve as a hub and spoke model enabling partnerships with local general practices to provide integrated reproductive health care based on local needs.

### Towards a health systems approach to abortion provision

As early MTOP provision is largely only available in the private(for profit) sector, consideration of the role of the public system in abortion services is necessary to ensure an integrated approach that facilitates access for disadvantaged women, all options for abortion and support for GPs. In Australia the newly formed Primary Health Care Networks are in a strong position to provide the necessary infrastructure to better support the role of general practice and coordinate the activities of health professionals, Local Health Districts and nongovernment organisations. These independent organisations are aligned with Local Hospital Networks and with their mandate to improve the effectiveness of medical services, especially for priority populations, they can better coordinate abortion care across the primary and public health systems [31].

### Limitations

Self-selection bias was a possibility in this study. GPs providers who agreed to participate may have been more likely to have strong views about abortion provision and the challenges they face in provision or referral, and those who did not take part may have been more likely to find the process straightforward. The ambivalence of some interviewees and similarities in challenges others faced suggests this was not the case. Furthermore, no GP who refused to refer women took part, although the views of conscientious objectors may have contributed further to the overall picture of access to abortion and informed strategies to address this. We interviewed GPs from the state of NSW, and findings may not be applicable to other states where the legislative and policy environments are different. These may be considered limitations of this study. However, this study is the first Australian study to include both MTOP provider and non-provider GPs, and therefore gives a comprehensive picture of the reasons why GPs do and do not provide MTOP as well as the challenges faced by active or potential providers.

## Conclusion

Although there are few GPs providing MTOP in NSW, GPs in this study were largely either motivated to provide MTOP or were supportive of referring women to abortion services. The GP participants identified a number of challenges and opportunities to improve practice in this area. These centred on the need for local strategies for women and healthcare professionals to improve access to abortion services including timely referral pathways to specialist care, support for follow-up care, improved access to MTOP medication and associated tests and the development of peer support networks and mentoring. There is need for state policy and referral pathways to support local MTOP provision. A publically funded hub and spoke model could link GPs with services to provide women with locally available comprehensive abortion care. These structural interventions require change at the professional and institutional levels as well as a whole of health system approach to improve the supply of MTOP GP providers and public sector provision.

## Abbreviations

GP: General practitioner
MTOP: Medical termination of pregnancy
NSW: New South Wales
SRH: Sexual and reproductive health
